# Expression of MHC products and leucocyte differentiation antigens in gynaecological neoplasms: an immunohistological analysis of the tumour cells and infiltrating leucocytes.

**DOI:** 10.1038/bjc.1985.227

**Published:** 1985-10

**Authors:** A. Ferguson, M. Moore, H. Fox

## Abstract

**Images:**


*Br. J. Cancer* (1985), **52**, 551–563

### Expression of MHC products and leucocyte differentiation antigens in gynaecological neoplasms: An immunohistological analysis of the tumour cells and infiltrating leucocytes

A. Ferguson[1], M. Moore[2] & H. Fox[3]

*Departments of [1]Obstetrics and Gynaecology; [3]Reproductive Pathology, St. Mary's Hospital, Manchester M13 0JH; [2]Department of Immunology, Paterson Laboratories, Christie Hospital and Holt Radium Institute, Manchester M20 9BX, UK.*

**Summary** Monoclonal antibodies directed against monomorphic determinants of Class I and Class II products of the major histocompatibility complex (MHC) and against leucocyte differentiation antigens were used in an indirect immunoperoxidase technique to compare their expression in normal and malignant disease of the ovary, cervix and endometrium. MHC Class I products, strongly expressed on normal ovarian epithelium, were uniformly absent from 7/8 ovarian carcinomas of varying histology. Lack of Class I expression was also a feature of 6/10 cervical carcinomas and of 4/8 endometrial carcinomas, in comparison with their repsective normal tissues. Relative to normal tissue epithelium MHC Class II products, could be either lost or gained, the pattern of expression being either uniform or heterogeneous.

Leucocytes were sparse in normal ovary but more numerous in cervix and endometrium. In tumours, with few exceptions, they were abundant, though usually confined to the stroma. T cells, largely of cytotoxic/suppressor (OKT8) phenotype, tended to predominate though in some tumours, particularly cervical carcinoma, large numbers of macrophages and to a lesser extent, B cells, were sometimes detected. By contrast, leucocytes of natural killer (NK) phenotype were virtually non-existent in any tumour or normal tissue.

The ingress of leucocytes into gynaecological neoplasms does not appear to be a random event and may be evoked by an immune response against tumour-associated antigens. However, the relationship between *in situ* mononuclear cell infiltration and MHC expression on epithelial tumour cells is complex and remains to be elucidated.

It is now widely recognised that the biological behaviour of tumours, which may differ markedly for neoplasms of similar histopathological category and grade, is determined to a significant extent by interactions with neighbouring cells and by their environment in general. Attempts to unravel the critical factors in these complex *in vivo* interactions have, in part, focussed on the relationship between leucocyte infiltration and prognosis, for which, there exists, at least for some neoplasms, a positive correlation (Underwood, 1974; Ioachim, 1976) as well as upon the anti-tumour properties *in vitro* of inflammatory cells recovered from disaggregated neoplasms (see Haskill, 1982; Moore, 1984; Vose & Moore, 1985).

In the study of human tumours, only a limited examination of the many parameters involved in leucocyte ingress is presently feasible mainly because the antigens expressed by these neoplasms have only been partially defined. Of theoretical relevance to host recognition of tumour cells in this context, is the expression of products of the major histocompatibility complex (MHC). Class I (HLA-A, B, C) antigens are found on virtually all normal epithelial cells, and their recognition is essential for the killing of virus-infected target cells (McMichael, 1978) by cytotoxic T lymphocytes and possibly of tumour cells also. MHC Class II products were until recently, considered to be restricted to cells of the immune system, including B lymphocytes, macrophages, vascular endothelial, dendritic and other antigen presenting cells, as well as activated T lymphocytes (Ko *et al.*, 1979). Now in addition, certain epithelia and a significant number of non-lymphoid neoplasms including those of breast, colorectal carcinoma and malignant melanoma are known to display Class II molecules (Natali *et al.*, 1981; Thompson *et al.*, 1982; Whitwell *et al.*, 1984; Daar & Fabre, 1983; Rognum *et al.*, 1983). Currently there is interest in whether this property is a requirement for the induction of autologous lymphoproliferative responses by tumour antigens (Guerry *et al.*, 1984).

The availability of McAbs to the framework determinants of MHC Class I and Class II products as well as to leucocyte differentiation antigens permits simultaneous immunohistochemical analysis

Correspondence: M. Moore.
Received 15 April 1985; and in revised form, 9 July 1985.

© The Macmillan Press Ltd., 1985

of the MHC status of tumour cells and the *in situ* host response in a manner which has hitherto proved impossible using conventional histological techniques. In this study we extend this approach, previously applied in this laboratory to breast and colorectal carcinomas (Whitewell *et al.*, 1984; Csiba *et al.*, 1984) to a comparison of neoplasms of the cervix, endometrium and ovary, with their normal tissue counterparts.

### Patients and methods

### *Patients*

Non-neoplastic cervical and endometrial tissues were obtained from patients (aged 35–53 years) undergoing total hysterectomy. The normality of the tissues and their hormonal status were confirmed by histological screening.

Normal ovarian tissue was generally obtained from patients of menopausal age undergoing hysterectomy at the time of the menopause, when bilateral salpingo-oophorectomy is often routinely carried out at the same time. The indications for hysterectomy operations in this group were usually abnormal bleeding or fibroids and the ages of the patients ranged from 45–76 years. Specimens judged histologically to have pathological changes were discarded.

Endometrial carcinomas were likewise obtained from hysterectomy specimens and cervical neoplasms from Wertheims hysterectomy specimens and on one occasion by cervical biopsy. Patients with cervical and endometrial carcinoma were aged from 31–77 and 52–76 years respectively. Ovarian tumours were obtained at laparotomy. The age range of these patients was 50–69 years. Histopathological data on each specimen are incorporated into Tables II, III and IV.

### *Processing of specimens*

Tissues were snap frozen within one hour of surgical removal and stored over liquid nitrogen. Serial sections (5–10 $\mu$m) were cut, mounted on glass slides and air-dried. Each tissue was sampled at three different planes, all three being mounted on the same slide. When necessary, slides could be stored at $-20$°C under dessicated conditions for up to one month. Prior to staining, slides were returned to room temperature, fixed in acetone for 5 min, air-dried and immersed in 20% new born calf serum in PBS, pH 7.5. Thereafter sections were treated according to procedures previously described from this laboratory (Whitwell *et al.*, 1984). Briefly, sections were incubated in the monoclonal antibody (McAb) first layer, washed and then exposed to diluted horse radish peroxidase-conjugated rabbit anti-mouse Ig (Dako) containing normal human serum. The peroxidase reaction was developed in diaminobenzidine containing freshly added $H_2O_2$, the sections washed and counterstained in Gills No. 2 Haemalum and mounted. The specificity of the McAbs was systematically checked on sections of palatine tonsil, lymph node or spleen, against which new batches of reagents were also titrated. No attempt was made to abolish endogenous staining, which when present, was readily distinguishable from specific immunostaining. Tissue reactions were scored semi-quantitatively (see footnote to Table II) as previously described (Whitwell *et al.*, 1984). In most instances, morphometric analyses were precluded by the intra-tissue variation across a given section.

### *Monoclonal antibodies (McAbs)*

With the exception of the B73.1 reagent, details of the McAbs used in this study have been given previously (Whitwell *et al.*, 1984; Csiba *et al.*, 1984) and are summarised in Table I.

### Results

Clinicopathological and immunohistological data on serial sections from normal tissues and malignant tumours are summarised in Tables II (ovary), III (cervix) and IV (endometrium) and illustrative examples of specific immunostaining are given in the Figures.

A feature common to all tissues examined was the paucity of cells of NK phenotype. Only the occasional B73.1$^+$ or Leu 7 (HNK1)$^+$ cell was detected in a small minority of some 60 tissues examined. This was so in malignant tumours despite the often large increase in leucocytes over normal tissues. B73.1$^+$ cells were routinely detectable in normal human spleen and Leu 7 (HNK1)$^+$ cells in the germinal centres of lymphoid tissue (posotive controls).

*Normal ovary* MHC Class I products (reactive with 2A1) were invariably detectable on the germinal epithelium, follicle lining cells and endothelial cells and with much less staining intensity on the scattered stromal cells.

MHC Class II products (reactive with TDR 31.1) were also detectable on endothelial and follicle lining cells but absent from epithelial and connective tissue cells. Few DR$^+$ leucocytes were seen. 2D1$^+$ leucocytes were evenly but sparsely distributed throughout the ovarian stroma. Comparison of UCHT1 staining with that of

**Table I** Monoclonal antibodies (McAbs) to major histocompatability complex (MHC) products and leucocyte differentiation antigens

| McAb (*murine*)[b] | *Ig class/ subclass* | *Specificity* | *Origin* | *References* |
|---|---|---|---|---|
| 2A1 | IgG1 | anti HLA Class I (HLA-A, -B, -C) | P.C.L. Beverley | Beverley, 1980. |
| TDR31.1 | IgG1 | anti HLA Class II (HLA-DR) | J. Bodmer | DeKretser *et al.*, 1982 |
| 2D1 | IgG1 | Common leucocyte antigen | P.C.L. Beverley | Beverley *et al.*, 1980 |
| UCHT1 | IgG1 | T cell receptor associated molecule (gp 19,000) | P.C.L. Beverley | Callard *et al.*, 1981 |
| (This reagent possesses identical reactivity to OKT3). | | | | Kung *et al.*, 1979 |
| OKT4 | IgG2b | T helper/inducer subset | Ortho | Kung *et al.*, 1979; Reinherz *et al.*, 1979*a*, *b* |
| OKT8 | IgG2a | T cytotoxic/suppressor subset | Ortho | Reinherz *et al.*, 1980 |
| OKMI | IgG2b | C3bi receptor (reactive with monocytes/macrophages; large granular lymphocytes) | Ortho | Breard *et al.*, 1980 |
| Leu 7 (HNK1) | IgM | Large granular lymphocytes (LGL): T cell subset | C.M. Balch | Abo & Balch, 1981; Abo *et al.*, 1982*a*. |
| MAS020 | IgG1 | B cells[a] | Sera Lab. | J. Habeshaw[a] (personal communication) |
| B73.1 | IgG1 | Large granular lymphocytes (LGL) (FcγR) | G. Trinchieri | (Perussia *et al.*, 1983*a*, *b*) |

[a]Reactive with inter- and intra-follicular B cells (determined on palatine tonsils) and probably reactive with a polymorphic B cell determinant; [b]Second layer reagent: Horse radish peroxidase conjugated rabbit anti-mouse Ig (Dako).

MAS020 suggested that, although few, the numbers of T and B lymphocytes were approximately even, with those of the OKT8 subset more frequently detectable than those of the OKT4 subset. Leucocytes were mainly associated with blood vessels and follicles, and included some OKM1$^+$ cells in a quarter of the tissues examined. In one ovary containing a corpus luteum, the luteal cells were stained with OKM1 and MAS020. Interestingly, the epithelial cells of one benign tumour (a serous cystadenomena) were positive for both Class I and Class II products.

*Ovarian carcinoma (Table II)* Seven ovarian carcinomas examined failed to express MHC Class I products regardless of histological type or clinical grade but stromal cells expressed the antigens strongly. However, not all histological categories of ovarian neoplasm were represented. Contrariwise, 4/8 carcinomas expressed DR antigens (Figures 1 and 2) where none were detectable in normal epithelium. Staining was clearly associated with the malignant cells where it was either uniform (Figure 1) or heterogeneous (Figure 2) although DR$^+$ leucocytes (? B cells, monocytes, activated T cells) were also detected in the stroma of many tumours. Seven tumours were characterised by a massive influx of 2D1$^+$ leucocytes which was unrelated to the degree of necrosis. While these were most numerous in the stroma, in 6/8 specimens leucocytes had also penetrated the tumour mass.

Comparison of staining with the various McAbs indicated that the leucocyte stroma consisted of T cells in excess of B cells and monocytes/macrophages. OKT4$^+$ cells were detectable in 6/8 samples and OKT8$^+$ cells in 8/8. Overall, OKT8$^+$ cells obviously exceeded OKT4$^+$ cells in 3/8 cases. All tumours contained OKM1$^+$ cells, mostly in the stroma, but also in areas of tumour necrosis.

*Normal cervix* MHC Class I antigens were strongly expressed on the lower one-third to one half of the squamous epithelium (Figure 3). In 10/14 samples endocervical glands were present, which were Class I positive. There was no obvious correlation with the hormonal status of the women. MHC Class II antigens were indetectable on epithelial cells but almost all tissues revealed a few DR$^+$ leucocytes scattered at the base of the squamous epithelium and around the squamo-columnar junction and endocervical glands. Seven of 10 samples which contained endocervical glands exhibited Class II staining of the glandular cells which was again apparently unrelated to the hormonal status of the women.

2D1$^+$ leucocytes were present in moderate numbers in 13/13 specimens. They were most

**Table II** Summary of specific immunostaining with McAbs to MHC products and leucocyte differentiation antigens of normal ovary (8 pts) and ovarian carcinoma (8 pts)

| | | | Epithelial cells[b] | | Leucocytes[a] | | | | | | |
|---|---|---|---|---|---|---|---|---|---|---|---|
| *Patient* | *Histology* | *Age* | *2A1* | *TDR31.1* | *2D1* | *UCHT1* | *OKT4* | *OKT8* | *OKM1* | *TDR31.1* | *MAS020* |
| (X8) | Normal ovary (salpingo-oophorectomy specimens) | 35–76 | + | – | + | +/– | +/– | +/– | +/– | +/– | + |
| JR | Well diff. endometrioid adenocarcinoma | 50 | – | + | +++ | ++ | + | + | + | +/– | + |
| PP | Very undiff. adenocarcinoma with serosal metastases | ? | – | – | ++++ | +++ | + | +++ | ++ | + | ++ |
| DH | Well diff. endometrioid adenocarcinoma. No metastases | 63 | – | – | +/– | – | – | +/– | +/– | + | – |
| MB | Poorly diff. adenocarcinoma of indeterminate cell origin. No metastases | 50 | – | +/– | ++++ | +++ | ++ | + | + | +/– | +++ |
| MP | Moderately well differentiated serous cystadenocarcinoma with metastases. | 69 | +/– | – | ++ | ++ | + | + | ++ | + | + |
| EC | Adenocarcinoma of mixed serous and endometrioid pattern Well diff. in some areas and poorly diff. in others with metastases to the R. fallopian tube | 64 | – | + | ++++ | ++ | +/– | ++ | +++– | ++ | ++ |
| HB | Moderately differentiated adenocarcinoma | 60 | – | + | ++ | +/– | +/– | +/– | +++ | ++ | ++ |
| AF | Well-differentiated endometrioid adenocarcinoma | 52 | – | – | ++++ | ++ | + | ++ | +/– | +/– | + |

[a]Staining reactions are scored on a semi-quantitative scale from 4+ (many cells stained) to – (no cells stained) for the leucocytic infiltrate. (B73.1 and Leu 7 (HNK1) omitted on account of paucity of positive cells). [b]Staining reactions with anti-HLA antibodies are denoted by + (homogenous staining); – (no staining) and +/– (heterogeneous staining).

**Figure 1** Ovarian endometrioid adenocarcinoma stained for MHC Class II (TDR 31.1) and showing strong and uniform expression on the tumour cells, but not on the supporting stroma. Counterstained with haematoxylin (×220).

**Figure 2** Poorly differentiated ovarian adenocarcinoma stained for MHC Class II (TDR 31.1) showing heterogeneity of expression among the tumour cells. The supporting stromal tissue is negative but contains some $DR^+$ leucocytes. Counterstained with haematoxylin (×220).

**Figure 3** Normal cervical squamous epithelium stained for MHC Class I (2A1) showing pronounced uniform expression in the lower one-third to one-half of the squamous epithelium. Scattered leucocytes and vessel lining cells in the sub-epithelial region are also positive. Counterstained with haematoxylin (×220).

**Figure 4** Normal cervical squamous epithelium stained for T lymphocytes (UCHT1) showing numerous positive cells at the base and in the lower half of the squamous epithelium, and in the underlying connective tissue. Counterstained with haematoxylin (×220).

numerous at the base of the squamous epithelium at the squamo-columnar junction and around endocervical glands. They were also found scattered throughout the lower half of the squamous epithelium. Most of the $2D1^+$ populations were also $UCHTI^+$ indicating a predominance of T cells (Figure 4) but not to the exclusion of other cell types. $OKT8^+$ cells were more consistently demonstrable (12/12 tissues) and numerically superior to OKT4 cells (detected in 8/13 tissues). Eleven of 12 tissues contained $MAS020^+$ cells, albeit in low numbers around endocervical glands which in most instances were also stained with this reagent. $OKM1^+$ cells were equally sparsely represented in 9/14 tissues, with a slight excess at the squamo-columnar junction.

*Cervical carcinoma* (*Table III*) Six of 10 cervical carcinomas failed to express MHC Class I antigens (Figure 5). One (patient JS, Table III) exhibited uniform 2A1 staining and three others (JC, SC, JH) heterogeneous staining. Two carcinomas (patients JC, SC) revealed DR positivity of the tumour cells.

All 10 carcinomas were massively infiltrated with $2D1^+$ leucocytes which had a predominantly stromal localisation, though in 8 neoplasms there was also significant penetration of the tumour mass. Again, $2D1^+$ cell infiltration was not related to tumour necrosis. Comparison with UCHTI staining indicated that the majority of $2D1^+$ cells were usually T cells with the OKT8 subset clearly exceeding the OKT4 subset in 5/10 cases. However, B cells ($MAS020^+$) were present in the leucocytic

**Table III** Summary of specific immunostaining with McAbs to MHC products and leucocyte differentiation of normal cervix (13 pts) and cervical carcinoma (10 pts)

| | | | *Epithelial cells*[b] | | *Leucocytes*[a] | | | | | | |
|---|---|---|---|---|---|---|---|---|---|---|---|
| *Patient* | *Histology* | *Age* | *2A1* | *TDR31.1* | *2D1* | *UCHT1* | *OKT4* | *OKT8* | *OKM1* | *TDR31.1* | *MAS020* |
| (X13) | Normal cervix (hysterectomy specimens) | 35–53 | + | − | +++ | +++ | + | + | +/− | + | +/− |
| JC | Invasive keratinising well diff. squamous cell ca. +ve lymph node metastases. | 44 | +/− | + | ++++ | +++ | +++ | +++ | ++ | + | ++++ |
| LG | Poorly diff. non-keratinising squamous cell ca. Post radiation recurrence. | 77 | − | − | ++ | + | +/− | + | +++ | ++ | +++ |
| JS | Invasive well diff. non-keratinising large cell squamous ca. | 31 | ++ | − | ++++ | +++ | ++ | ++ | ++ | + | +/− |
| DW | Well diff. adenocarcinoma. No metastases. | 52 | − | − | ++++ | +++ | ++ | + | ++++ | +++ | +++ |
| WR | Large cell focally keratinising mod. well diff. invasive squamous cell ca. (Biopsy). | 68 | − | − | +++ | ++ | +/− | + | ++ | + | − |
| SC | Well diff. adenocarcinoma +ve lymph node metastases. | ? | +/− | + | ++ | ++ | +/− | ++ | ++ | + | +/− |
| JH | Well diff. keratinising squamous cell ca. No metastases. | 40 | +/− | − | ++++ | ++ | − | ++ | − | +/− | +/− |
| MM | Infiltrating non-keratinising poorly diff. squamous cell ca. +ve lymph node metastases. | 31 | − | − | +++ | +++ | − | ++ | + | + | +++ |
| MJ | Undifferentiated carcinoma +ve lymph node metastases. | 31 | − | − | ++++ | ++++ | ++ | +++ | +/− | + | +++ |
| CO'N | Extensive mod. diff. squamous cell ca. No metastases but lymphatic penetration. | 34 | − | − | +++ | +++ | +/− | + | ++ | + | − |

[a,b]see footnote to **Table II**.

**Figure 5** Cervical carcinoma stained for MHC Class I (2A1) showing absence from the tumour cells in contrast with positive expression on stromal and endothelial cells. Counterstained with haematoxylin (×220).

**Figure 6** Cervical carcinoma stained for monocytes/macrophages (OKM1) showing numerous positive cells within the tumour mass (×220).

stroma in 8/10 specimens (in relatively large numbers in five of these). In two cases this reagent also stained the tumour cells. Nine of 10 samples contained substantial numbers of OKM1 cells mostly confined to the stroma but sometimes in close juxtaposition to tumour cells (Figure 6). Staining with OKM1 was also marked at the necrotic centre of three neoplasms.

*Normal endometrium* Cells comprising the endometrial glands, myometrial tissue, endothelium and the stromal component of the endometrium were uniformly $2A1^+$ and in 6/13 cases the endometrial glands were also Class II positive. This latter property was not related to the hormonal status of the patients.

$2D1^+$ leucocytes were present in moderate numbers in all 13 tissues examined, a small proportion of which were $DR^+$. These were more numerous in the endometrium than in the underlying myometrium and tended to cluster around the endometrial glands. The leucocytes consisted predominantly of T cells of which $OKT8^+$ cells were more consistently detected (12/12 cases) than $OKT4^+$ cells (9/13 cases). Ten of 12 specimens contained low numbers of B cells and there were also few $OKM1^+$ cells. Some normal endometrial glands were stained with Leu 7 (HNK1) and MAS020. There was little apparent quantitative variation in the number and subtype of leucocytes during the varying phases of the menstrual cycle.

*Endometrial carcinoma* (*Table IV*) Four of 8 tumours were entirely lacking in MHC Class I antigen and expression in 4 others was heterogeneous. Stromal tissue was strongly positive in all cases. There was no correlation between antigen expression and tumour grade. Only 2/8 tumours (patients BC and FH) expressed DR antigens on the epithelial cells (cf. Figure 7); these tumours were well differentiated and the pattern of staining was heterogeneous.

$2D1^+$ cells were present in large numbers in all 8 tumours examined. These were especially numerous at the junctional areas between tumour and normal tissue and in the tumour stroma. Five samples showed significant penetration of the tumour mass.

Most of the $2D1^+$ leucocytes were T cells. Again, in 4/8 samples $OKT8^+$ cells were numerically superior to $OKT4^+$ cells. In this series, lymphocytes which had penetrated the tumour mass were predominantly of OKT8 phenotype (Figure 8) and

**Figure 7** Endometrial adenocarcinoma stained for MHC Class II (TDR 31.1) showing absence from the tumour cells but positive expression on vessels and leucocytes within the tumour mass. Also shown is an area of architectural atypia adjacent to the tumour, the staining pattern of which is heterogeneous. Counterstained with haematoxylin (×220).

**Table IV** Summary of specific immunostaining with McAbs to MHC products and leucocyte differentiation antigens of normal endometrium (13 pts) and endometrial carcinoma (8 pts)

| | | | *Epithelial cells*[b] | | *Leucocytes*[a] | | | | | | |
|---|---|---|---|---|---|---|---|---|---|---|---|
| *Patient* | *Histology* | *Age* | *2A1* | *TDR31.1* | *2D1* | *UCHT1* | *OKT4* | *OKT8* | *OKM1* | *TDR31.1* | *MAS020* |
| (X13) | Normal endometrium (hysterectomy specimens) | 35–50 | ++ | + | +++ | +++ | + | ++ | +/− | + | +/− |
| AF | Well diff. adenocarcinoma infiltrating inner 1/3rd myometrium. No evidence of vascular involvement. | 71 | − | − | ++++ | +++ | ++ | +++ | +++ | ++ | ++ |
| BC | Very well diff. adenocarcinoma (Grade I) penetrating inner 1/3rd myometrium. | 53 | +/− | +/− | +++ | ++ | ++ | ++ | +++ | ++ | + |
| LW | Poorly diff. carcinoma in a polyp. None in myometrium. | 74 | +/− | − | +++ | ++ | ++ | ++ | +/− | − | + |
| FH | Mod. well diff. adenocarcinoma (Grade II) infiltrating inner ¼ myometrium. | 60 | ++ | +/− | +++ | +++ | ++ | +++ | +++ | ++ | − |
| CM | Mod. well diff. adenocarcinoma (Grade II) extending to outer ½ of myometrium but poorly diff. at infiltrating margin. | 55 | − | − | ++++ | +++ | +++ | +++ | +++ | ++ | +++ |
| LH | Well diff. endometrioid adenocarcinoma infiltrating the entire myometrial vasculature and extending onto the serosal surface. | 74 | − | − | +++ | ++ | ++ | ++ | ++ | + | +/− |
| JD | Very well diff. adenocarcinoma pentrating inner 1/3rd of myometrium. | 76 | +/− | − | ++++ | ++ | + | ++ | +/− | +/− | + |
| RC | Well diff. adenocarcinoma confined to inner 1/3rd of myometrium (Grade I). | 52 | − | − | ++++ | ++ | ++ | +++ | +++ | ++ | +/− |

[a,b]See footnote to **Table II.**

**Figure 8** Well differentiated adenocarcinoma of endometrium showing heavy ingress of suppressor/cytotoxic ($OKT8^+$) T cells. Counterstained with haematoxylin (×220).

**Figure 9** Adenocarcinoma of endometrium stained for MHC Class II (TDR 31.1) showing numerous $DR^+$ stromal leucocytes of lymphocyte and macrophage morphology, and $DR^-$ epithelial cells. Counterstained with haematoxylin (×220).

$DR^+$ leucocytes which comprised macrophages as well as lymphocytes (Figure 9). B cells were also represented in moderate numbers in the stroma, but were exceeded by cells of $OKM1^+$ phenotype, which were also detectable in the tumour mass (5/8 cases). In 4 of the 8 neoplasms, apparent cross-reactivity with MAS020 of the most differentiated tumour cells was in evidence. In addition, some fibrous septal areas between endometrial tumour masses were stained with Leu 7 (HNK1).

### Discussion

While several tumour-associated antigens of gynaecological neoplasms have been detected serologically (Bast *et al.*, 1983; Bhattacharya *et al.*, 1982; Masuho *et al.*, 1984) their ability to evoke immune responses in the autochthonous host is unknown. Accordingly, attention was focussed on the MHC products of tumour cells which are likely to be involved in immune induction and tumour cell recognition and the nature of the *in situ* inflammatory cells as defined by monoclonal antibodies. In respect of these properties this study has disclosed a number of potentially important differences between malignant gynaecological tumours and their normal tissue counterparts. It is preliminary to the extent that the panel of McAbs was limited as were the numbers of tumours examined of each of the major types.

Malignant epithelial cells were frequently MHC Class I negative, under conditions where the stroma was strongly positive. The difference from normal tissue was most marked in the case of cervical and ovarian carcinoma where the majority of tumours were negative. A trend towards Class I negativity among endometrioid ovarian neoplasms was reported by Kabawat *et al.* (1983). Our failure to encounter Class I positive tumours in our smaller series is thus probably a reflection of the predominance of these histological types rather than of differential staining sensitivity. To our knowledge, the HLA Class I status of cervix and endometrial carcinomas and their normal tissue counterparts has not been previously reported (cf. Daar *et al.*, 1984*a*). Depressed or heterogeneous expression of MHC Class I products, as detected by immunohistological procedures, is a property of some tumours of diverse histogenic derivation including breast (Fleming *et al.*, 1981; Bhan & Des Marais, 1983; Rowe & Beverley, 1984; Whitwell *et al.*, 1984) and to a lesser extent, colorectal carcinoma (Csiba *et al.*, 1984). Other neoplasms, notably malignant melanoma (Ruiter *et* al., 1982) and certain histological categories of ovarian carcinoma (Kabawat *et al.*, 1983) appear to express Class I antigens with greater consistency, a phenomenon which is apparently related to the predominantly T cell infiltrate which is a feature of these tumours.

The level of the failure of primary tumours to express Class I antigens has yet to be elucidated. The immunoperoxidase method as employed in this study is essentially a qualitative technique, so that levels of Class I expression below the threshold limits of detection, could conceivably occur, i.e. the deficit may be relative, rather than absolute.

As exemplified by genetic experiments with Daudi cells (Arce-Gomez *et al.*, 1978) some tumours may fail to express HLA due to lack of $\beta_2$ microglobulin ($\beta_2$m), the low molecular weight glycoprotein required to stablize the Class I heavy chain.

The extent to which the gynaecological neoplasms in this study synthesised and secreted $\beta_2$m was not ascertained. Failure to express MHC Class I antigens by malignant cells arising from HLA-positive tissues could, as Bodmer (1981) has advocated, be a change that could be selected for during tumour progression through the advantage of resistance to attack by cytolytic T cells. Such resistance might presumably only be an advantage to tumours that express tumour-associated antigens capable of evoking T cell immunity. However, this hypothesis may now require some revision since, at the clonal level, T cells lacking NK-like activity may recognise certain tumour types in an immunologically non-restricted fashion (De Vries & Spits, 1984). Thus, it may not be without significance in our study that OKT8$^+$ cell influx was not correlated with HLA Class I status.

It is also possible that lack of Class I determinants influences cell:cell interactions outside the immune system in a way, e.g. that favours the emergence of metastatic variants.

In common with other tissues, MHC Class II molecules, normally considered to be confined to cells of the immune system were frequently detectable on the glandular epithelia of normal cervix and endometrium (cf. Daar *et al.*, 1984*b*). The so-called 'aberrant expression' of Class II products on normal epithelial cells is associated with immunological, inflammatory as well as hormonal stimuli (Klareskog *et al.*, 1980; Lampert *et al.*, 1981; Mason *et al.*, 1981; Selby *et al.*, 1983) and similar stimuli could promote their expression on malignant epithelial cells.

In cervical and endometrial tumours MHC Class II expression appears to reflect the cellular origin of the neoplasm. On the other hand, tumours originating from apparently DR-negative epithelium (ovary) could acquire the ability to express Class II molecules in response to stimuli of the type mentioned above.

In both autoimmune and neoplastic diseases MHC Class II products on target cells are envisaged as a vehicle for the presentation of autoantigen to T helper/inducer lymphocytes on the assumption that the configuration of the particular Class II product fits that of the autoantigen (Londei *et al.*, 1984). In malignant melanoma, for instance, autologous lymphoproliferative responses can be induced by DR-positive tumour cells (Guerry *et al.*, 1984) but the extent to which similar responses are evoked against other categories of human malignancy has yet to be determined. In our series, the four DR$^+$ ovarian tumours appeared to show no excess T4 influx.

Analysis of the inflammatory infiltrates with McAbs to leucocyte differentiation antigens confirmed that infiltration occurs, to a very marked extent, in many gynaecological tumours. All categories of leucocyte reactive with McAbs in this series were increased in tumours in comparison with normal tissues. The greatest contrast was in carcinoma of the ovary, mainly because in the normal tissue leucocytes were few. In normal cervix where moderate numbers of leucocytes were consistently detectable and were mostly T cells, the greatest *difference* between normal and tumour tissues was the increased number of B cells and OKM1$^+$ macrophages. B cells were likewise present in moderate numbers in ovarian and endometrial carcinomas but not as numerous as in cervical carcinoma, where their numbers sometimes approached those of T cells. The extent to which these had differentiated into plasma cells was not easily discernible on cryostat sections.

Since OKM1$^+$ cells could not always be unequivocally identified as macrophages by morphological criteria, and $\sim$80% of the large granular lymphocyte (LGL) subset of peripheral blood is OKM1$^+$ (Ortaldo *et al.*, 1981), the LGL population was monitored in sections with two McAbs, one of which (B73.1) is reactive with virtually the entire LGL population (Perussia *et al.*, 1983*a*,*b*) and another (Leu 7; HNK1) which reacts with approximately 75% of LGL as well as a subset of T (suppressor) cells (Abo & Balch, 1981; Abo *et al.*, 1982*a*,*b*). B73.1$^+$ or Leu 7$^+$ cells were scarcely ever seen, indicating that the OKM1$^+$ population was probably wholly of monocyte/macrophage composition. In ovarian cancer, there is marked size variation and cytochemical heterogeneity among macrophages which is associated with a spectrum of activities from suppression to antibody-dependent cellular cytotoxicity (Haskill *et al.*, 1982*b*). However, gynaecological cancers, like those of breast, colon and lung (Watanabe *et al.*, 1983; Bhan & Des Marais, 1983; Whitwell *et al.*, 1984; Csiba *et al.*, 1984), contain few cells of natural killer phenotype.

This conclusion is in concordance with earlier functional and morphological data on a variety of tumours from several laboratories (Moore & Vose, 1981; Eremin *et al.*, 1981; Pizzolo *et al.*, 1984) and on ovarian cancer, in particular (Introna *et al.*, 1983; Kabawat *et al.*, 1983). The implications are that NK cells, which are maximally expressed in blood and spleen rarely extravasate and any direct anti-tumour activity is consequently very limited or non-existent.

The stimuli to leucocyte ingress into solid neoplasms are still largely unknown though the presumption remains that tumour antigenicity is a major, but certainly not the only factor. Whether human gynaecological neoplasms are immunogenic in the autologous host or not, the demonstration that the extravasation of leucocytes is not a random event might be relevant to host reponses of an immune nature. Although morphometric analysis of infiltrating populations is often complicated by marked heterogeneity in a given section, it is clear that some populations predominate over others. In many sections, there was little doubt that $T8^+$ cells exceeded $T4^+$ cells, indicative of a shift from the proportions normally present in peripheral blood, though perhaps to a lesser extent from those in patients with progressive or advanced disease (cf. McCluskey *et al.*, 1983). Similarly as already noted, in cervical carcinoma there was a significant ingress of B cells which was not consistent with random extravasation. However, it is possible that in certain carcinomas (e.g. endometrial), the association of $T8^+$ lymphocytes with epithelial cells is not indicative of a reaction to the tumour at all, but is rather a reflection of the normal relationship between the lymphocytes and epithelial cells in this tissue.

Functional studies showing depressed proliferative activity in T cells recovered from ovarian cancers implicated tumour inactivation as the cause, or failure of a particular subset to localise at the tumour site (Haskill *et al.*, 1982*a*). An alternative explanation might be that the predominant $T8^+$ subset contains functionally active suppressor cells (Vose & Moore, 1979). This possibility, which would theoretically favour tumour growth, might not be entirely unexpected in tumours which, apart from therapeutic intervention, have 'escaped' beyond recall as would doubtless have been the case for the tumours in this study. At this point, late in the progression of the lesions, the magnitude and type of cellular immune response is likely to have little *in vivo* operational significance to the advantage of the host.

This study was supported by grants from the Cancer Research Campign of Great Britain. We thank Dr Anne Simmonds, Glasgow Royal Infirmary, for contributing additional tissue to this study.

### References

ABO, T. & BALCH, C.M. (1981). A differentiation antigen of human NK and K cells identified by a monoclonal antibody (HNK-1). *J. Immunol.*, **127**, 1024.

ABO, T., COOPER, M.D. & BALCH, C.M. (1982*a*). Characterisation of HNK-1 (+) (Leu-7) human lymphocytes. I. Two distinct phenotypes of human NK cells with different cytotoxic capability. *J. Immunol.*, **129**, 1752.

ABO, T., COOPER, M.D. & BALCH, C.M. (1982*b*). Postnatal expansion of the natural killer and killer cell population in humans identified by the monoclonal HNK-1 antibody. *J. Exp. Med.*, **155**, 321.

ARCE-GOMEZ, B., JONES, E.A., BARNSTABLE, C.J., SOLOMON, E. & BODMER, W.F. (1978). The genetic control of HLA-A and B antigens in somatic cell hybrids: requirement for 2 microglobulin. *Tissue Antigens*, **11**, 112.

BAST, R.C., KLUG, T.L., ST. JOHN, E. & 9 others (1983). A radioimmunoassay using a monoclonal antibody to monitor the course of epithelial ovarian cancer. *N. Engl. J. Med.*, **308**, 883.

BHATTACHARYA, M., CHATTERJEE, S.K., BARLOW, J.J. & FUJI, H. (1982). Monoclonal antibodies recognising tumour-associated antigens of human ovarian mucinous cysadenocarcinomas. *Cancer Res.*, **42**, 1650.

BEVERLEY, P.C.L. (1980). Production and use of monoclonal antibodies in transplantation immunology. In *Transplantation and Clinical Immunology XI* Touraine *et al.* (eds) p.87. Excerpta Medica: Amsterdam.

BEVERLEY, P., LINCH, D. & DELIA, D. (1980). Isolation of human haematopoietic progenitor cells using monoclonal antibodies. *Nature*, **287**, 332.

BHAN, A.K. & DES MARAIS, E.L. (1983). Immunohistologic characterisation of major histocompatibility antigens and inflammatory cellular infiltrate in human breast cancer. *J. Natl Cancer Inst.*, **71**, 507.

BODMER, W.F. (1981). HLA structure and function: A contemporary view. *Tissue Antigens*, **17**, 9.

BREARD, J., REINHERZ, E.L., KUNG, P.C., GOLDSTEIN, G. & SCHLOSSMAN, S.F. (1980). A monoclonal antibody reactive with human peripheral blood monocytes. *J. Immunol.*, **124**, 1943.

CALLARD, R.E., SMITH, C.M., WOMAN, C., LINCH, D., CAWLEY, J.C. & BEVERLEY, P.C.L. (1981). Unusual phenotype and function of an expanded subpopulation of T cells in patients with haematopoietic disorders. *Clin. Exp. Immunol.*, **43**, 497.

CSIBA, A., WHITEWELL, H.L. & MOORE, M. (1984). Distribution of histocompatibility and leucocyte differentiation antigens in normal human colon and in benign and malignant colonic neoplasms. *Br. J. Cancer*, **50**, 699.

DAAR, A.S. & FABRE, J.W. (1983). The membrane antigens of human colorectal cancer cells: Demonstration with monoclonal antibodies of heterogeneity within and between tumours and of anomalous expression of HLA-DR. *Eur. J. Cancer Clin. Oncol.*, **19**, 209.

DAAR, A.S., FUGGLE, S.V., FABRE, J.W., TING, A. & MORRIS, P.J. (1984*a*). The detailed distribution of HLA-A,B,C antigens in normal human organs. *Transplantation*, **38**, 287.

DAAR, A.S., FUGGLE, S.V., FABRE, J.W., TING, A. & MORRIS, P.J. (1984*b*). The detailed distribution of MHC Class II antigens in normal human organs. *Transplantation*, **38**, 293.

DEKRESTER, T.A., CRUMPTON, M.J., BODMER, J.F. & BODMER, W.F. (1982). Two dimensional gel analysis of the polypeptides precipitated by a polymorphic HLA-DR1, 2, w6 monoclonal antibody: Evidence for a third locus. *Eur. J. Immunol.*, **12**, 600.

DE VRIES, J.E. & SPITS, H. (1984). Cloned human cytotoxic T lymphocyte (CTL) lines reactive with autologous melanoma cells. I. *In vitro* generation, isolation, and analysis to phenotype and specificity. *J. Immunol.*, **132**, 510.

EREMIN, O., COOMBS, R.R.A. & ASHBY, J. (1981). Lymphocytes infiltrating human breast cancers lack K-cell activity and show low levels of NK-cell activity. *Br. J. Cancer*, **44**, 166.

FLEMING, K.A., MCMICHAEL, A., MORTON, J.A., WOODS, J. & MCGEE, J.O.D. (1981). Distribution of HLA Class I antigens in normal human tissue and in mammary cancer. *J. Clin. Pathol.*, **34**, 779.

GUERRY, D., ALEXANDER, M.A., HERLYN, M.F. & 4 others. (1984). HLA-DR histocompatibility leukocyte antigens permit cultured human melanoma cells from early but not advanced disease to stimulate autologous lymphocytes. *J. Clin. Invest.*, **73**, 267.

HASKILL, J. (Ed.). (1982). *Tumour Immunity in Prognosis: The Role of Mononuclear Cell Infiltration*. Marcel Dekker Inc., New York.

HASKILL, S., KOREN, H., BECKER, S., FOWLER, W. & WALTON, L. (1982*a*). Mononuclear-cell infiltration in ovarian cancer: II Immune function of tumour and ascites-derived inflammatory cells. *Br. J. Cancer*, **45**, 737.

HASKILL, S., KOREN, H., BECKER, S., FOWLER, W. & WALTON, L. (1982*b*). Mononuclear-cell infiltration in ovarian cancer. III. Suppressor-cell and ADCC activity of macrophages from ascitic and solid ovarian tumours. *Br. J. Cancer*, **45**, 747.

IOACHIM, H.L. (1976). The stromal reaction of tumours: An expression of immune surveillance. *J. Natl Cancer Inst.*, **57**, 465.

INTRONA, M., ALLEVENA, P., BIONDI, A., COLOMBO, N., VILLA, A. & MANTOVANI, A. (1983). Defective natural killer activity within human ovarian tumours: Low numbers of morphologically defined effectors present *in situ*. *J. Natl Cancer Inst.*, **70**, 21.

KABAWAT, S.E., BAST, R.C. Jr., WELCH, N.R., KNAPP, R.C. & BHAN, A.K. (1983). Expression of major histocompatibility antigens and nature of inflammatory cellular infiltrate in ovarian neoplasms. *Int. J. Cancer*, **32**, 547.

KLARESKOG, L., FORSUM, U. & PETERSON, P.A. (1980). Hormonal regulation of expression of Ia-antigens on mammary gland epithelium. *Eur. J. Immunol.*, **101**, 958.

KO, H., FU, S.M., WINCHESTER, R.J., YU, D.T.Y. & KUNKEL, H.G. (1979). Ia determinants on stimulated human T lymphocytes. Occurrence on mitogen and antigen activated T cells. *J. Exp. Med.*, **150**, 246.

KUNG, P.C., GOLSTEIN, G., REINHERZ, E.L. & SCHLOSSMAN, S.F. (1979). Monoclonal antibodies defining distinctive human T cell surface antigens. *Science*, **206**, 347.

LAMPERT, I.A., SUITTERS, A.J. & CHISHOLM, P.M. (1981). Expression of Ia antigen on epidermal keratinocytes in graft-versus-host disease. *Nature*, **293**, 149.

LONDEI, M., LAMB, J.R., BOTTAZZO, G.F. & FELDMANN, M. (1984). Epithelial cells expressing aberrant MHC Class II determinants can present antigen to cloned human T cells. *Nature*, **312**, 639.

MASON, D.W., DALLMAN, M. & BARCLAY, A.M. (1981). Graft-versus-host disease induces expression of Ia antigens in rat epidermal cells and gut epithelium. *Nature*, **293**, 150.

MASUHO, Y., ZALUTSKY, M., KNAPP, R.C. & BAST, R.C. Jr. (1984). Interaction of monoclonal antibodies with cell surface antigens of human ovarian carcinomas. *Cancer Res.*, **44**, 2813.

MCCLUSKEY, D.R., ROY, A.D., ABRAN, W.P. & MARTIN, W.M.C. (1983). T lymphocyte subsets in the peripheral blood of patients with benign and malignant breast disease. *Br. J. Cancer*, **47**, 307.

MCMICHAEL, A.J. (1978). HLA restriction of human cytotoxic lymphocytes specific for influenza virus associated with HLA-A2. *J. Exp. Med.*, **148**, 1458.

MOORE, M. (1984). Tumour resistance and the phenomenon of inflammatory cell infiltration. In: *Handbook of Experimental Pharmacology* (Eds. Fox & Fox), Springer-Verlag, Berlin, **72**, 143.

MOORE, M. & VOSE, B.M. (1981). Extravascular natural cytotoxicity in man: Anti-K562 activity of lymph node and tumour infiltrating lymphocytes. *Int. J. Cancer*, **27**, 265.

NATALI, P.G., MARTINO, C.D., QUARANTA, V. & 4 others. (1981). Expression of Ia-like antigens in normal non-lymphoid tissues. *Transplantation*, **31**, 75.

ORTALDO, J.R., SHARROW, S.O., TIMONEN, T. & HERBERMAN, R.B. (1981). Determination of surface antigens on highly purified human NK cells by flow cytometry with monoclonal antibodies. *J. Immunol.*, **127**, 2401.

PERUSSIA, B., STARR, S., ABRAHAM, S., FANNING, V. & TRINCHIERI, G. (1983*a*). Human natural killer cells analysed by B73.1, a monoclonal antibody blocking Fc receptor functions. I. Characterisation of the lymphocyte subset reactive with B73.1. *J. Immunol.*, **130**, 2133.

PERUSSIA, B., ACUTA, O., TERHORST, C. & 4 others. (1983*b*). Human natural killer cells analysed by B73.1, a monoclonal antibody blocking Fc receptor functions. II. Studies of B73.1 antibody-antigen interaction on the lymphocyte membrane. *J. Immunol.*, **130**, 2142.

PIZZOLO, G., SEMENZATO, G., CHILOSI, M. & 5 others. (1984). Distribution and heterogeneity of cells detected by HNK-1 monoclonal antibody in blood and tissues in normal reactive and neoplastic conditions. *Clin. Exp. Immunol.*, **57**, 195.

REINHERZ, E.L., KUNG, P.C., GOLDSTEIN, G. & SCHLOSSMAN, W.F. (1979*a*). A monoclonal antibody with selective reactivity for functionally mature human thymocytes and all peripheral human T cells. *J. Immunol.*, **123**, 1312.

REINHERZ, E.L., KUNG, P.L., GOLDSTEIN, G. & SCHLOSSMAN, S.F. (1979*b*). Further characterisation of the human inducer T cell subset defined by monoclonal antibody. *J. Immunol.*, **123**, 2894.

REINHERZ, E.L., KUNG, P.C., GOLDSTEIN, G. & SCHLOSSMAN, S.F. (1980). A monoclonal antibody reactive with the human cytotoxic/suppressor T cell subset previously defined by a heteroantiserum termed TH2. *J. Immunol.*, **123**, 1301.

ROGNUM, T.O., BRANDZAEG, P. & THORUD, E. (1983). Is heterogeneous expression of HLA-DR antigens and CEA along with DNA-profile variations evidence of phenotypic instability and clonal proliferation in human large bowel carcinomas? *Br. J. Cancer*, **48**, 543.

ROWE, D.J. & BEVERLEY, P.C.L. (1984). Characterisation of breast cancer infiltrates using monoclonal antibodies to human leucocyte antigens. *Br. J. Cancer*, **49**, 149.

RUITER, D.J., BHAN, A.K., HARRIST, T.J., SOHER, A.J. & MIHM, M.C. Jr. (1982). Major histocompatibility antigens and mononuclear inflammatory infiltrate in benign nevomelanocytic proliferations and malignant melanoma. *J. Immunol.*, **129**, 2808.

SELBY, W.S., JANOSSY, G., MASON, D.Y. & JEWELL, D.P. (1983). Expression of HLA-DR antigen by colonic epithelium in inflammatory bowel disease. *Clin. Exp. Immunol.*, **53**, 614–618.

THOMPSON, J.J., HERLYN, M.F., ELDER, D.E., CLARK, W.H., STEPLEWSKI, Z. & KOPROWSKI, H. (1982). Expression of DR antigens in freshly frozen human tumours. *Hybridoma*, **1**, 161.

UNDERWOOD, J.C.E. (1974). Lymphoreticular infiltration in human tumours: Prognostic and biological implications: A review. *Br. J. Cancer*, **30**, 538.

VOSE, B.M. & MOORE, M. (1979). Suppressor cell activity of lymphocytes infiltrating human lung and breast tumours. *Int. J. Cancer*, **24**, 579.

VOSE, B.M. & MOORE, M. (1985). Human tumour-infiltrating lymphocytes – a marker of host response. *Semin. Haematol.*, **22**, 27.

WATANABE, S., SATO, Y., KODAMA, T. & SHIMOSATO, Y. (1983). Immunohistochemical study with monoclonal antibodies on immune response in human lung cancers. *Cancer Res.*, **43**, 5883.

WHITWELL, H.L., HUGHES, H.P.A., MOORE, M. & AHMED, A. (1984). Expression of major histocompatibility antigens and leucocyte infiltration in benign and malignant human breast disease. *Br. J. Cancer*, **49**, 161.


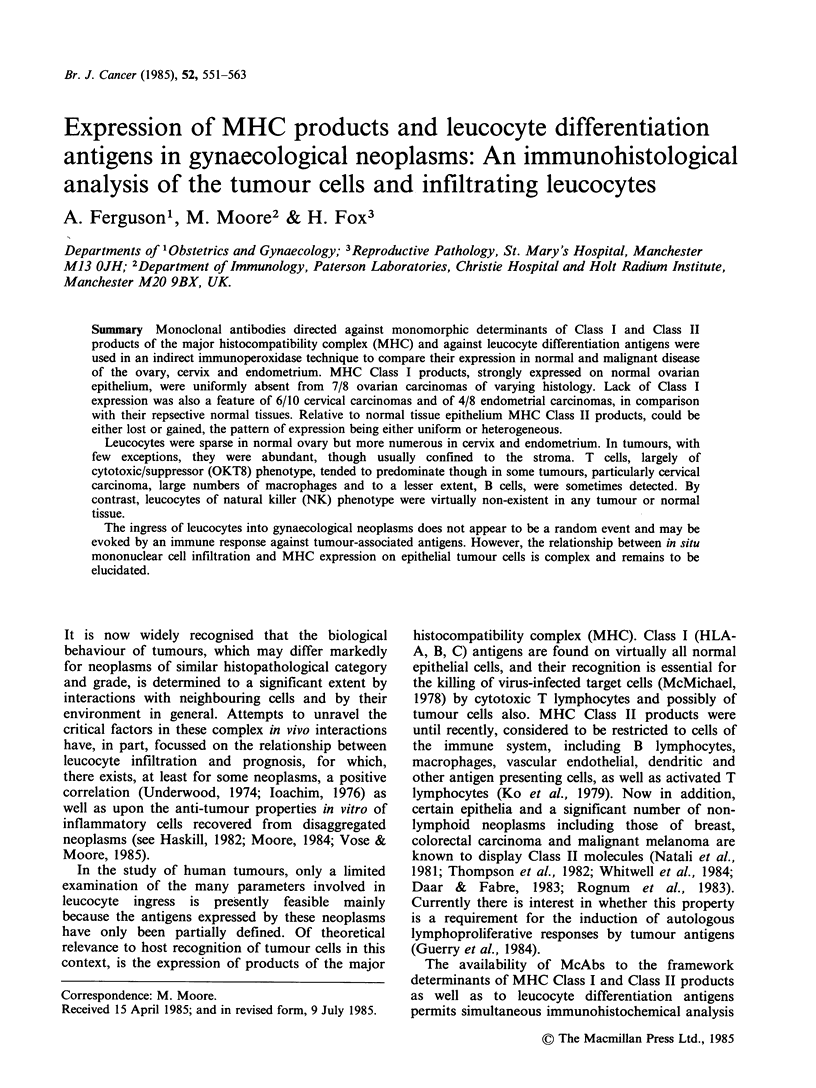

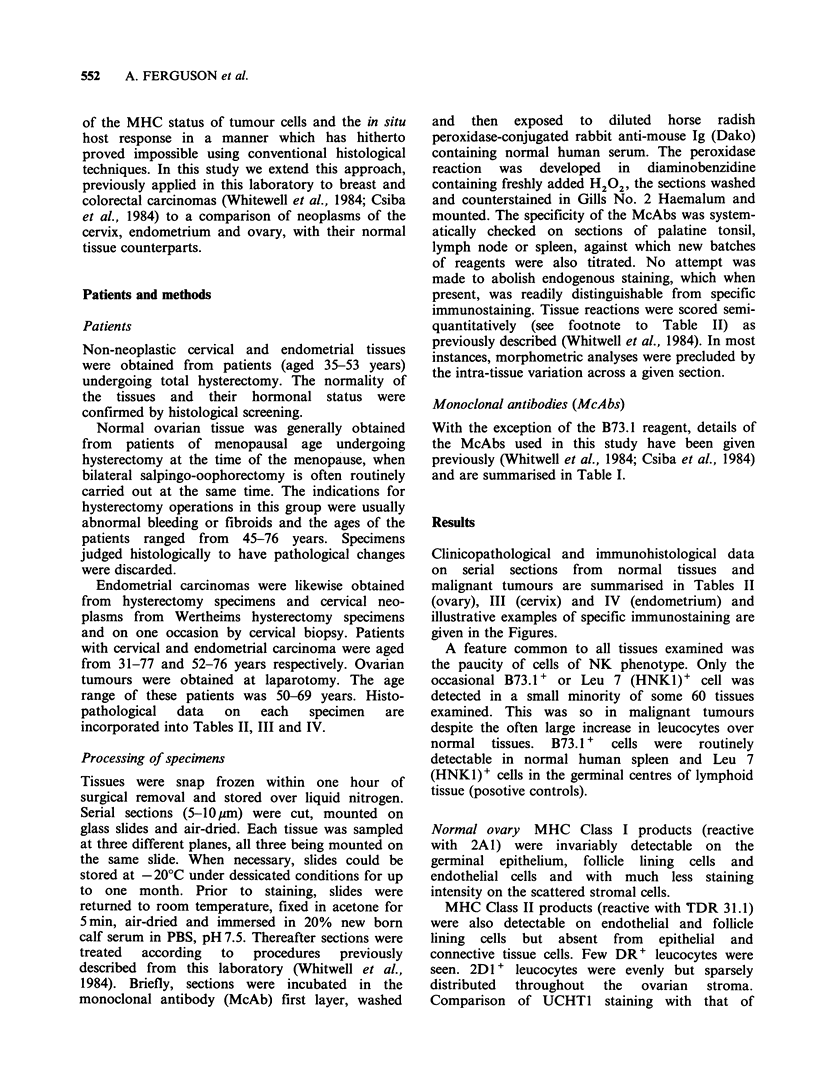

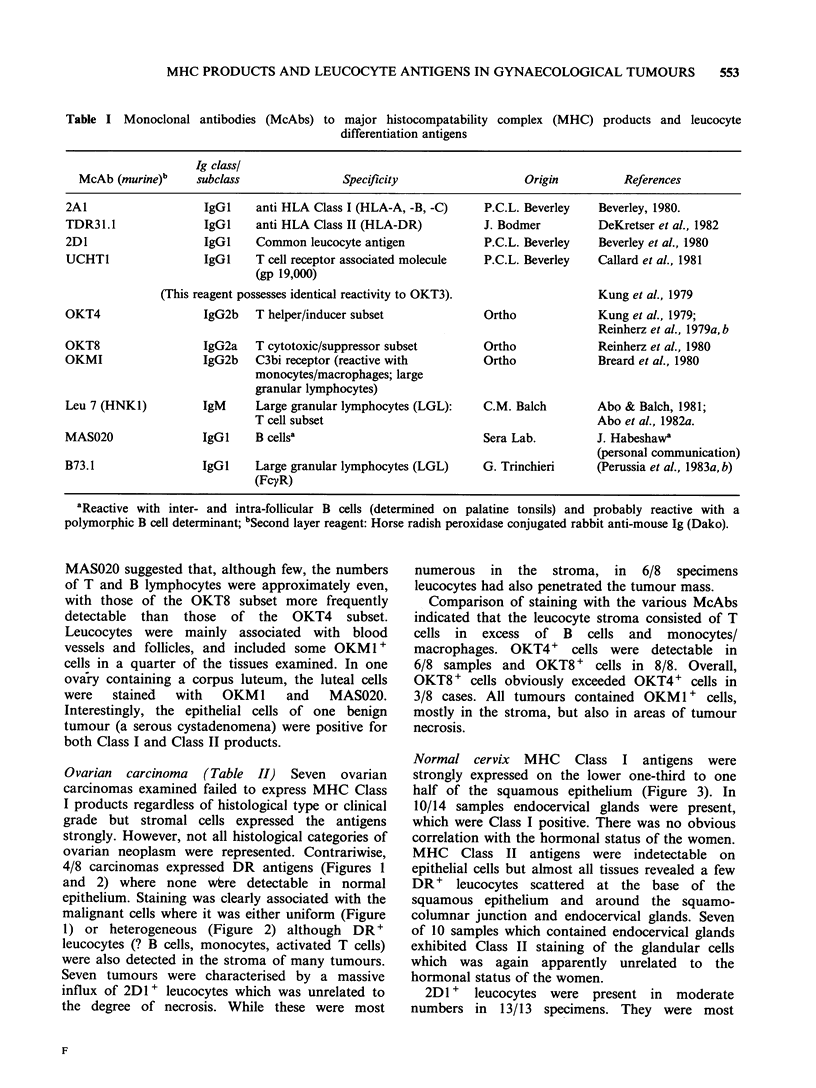

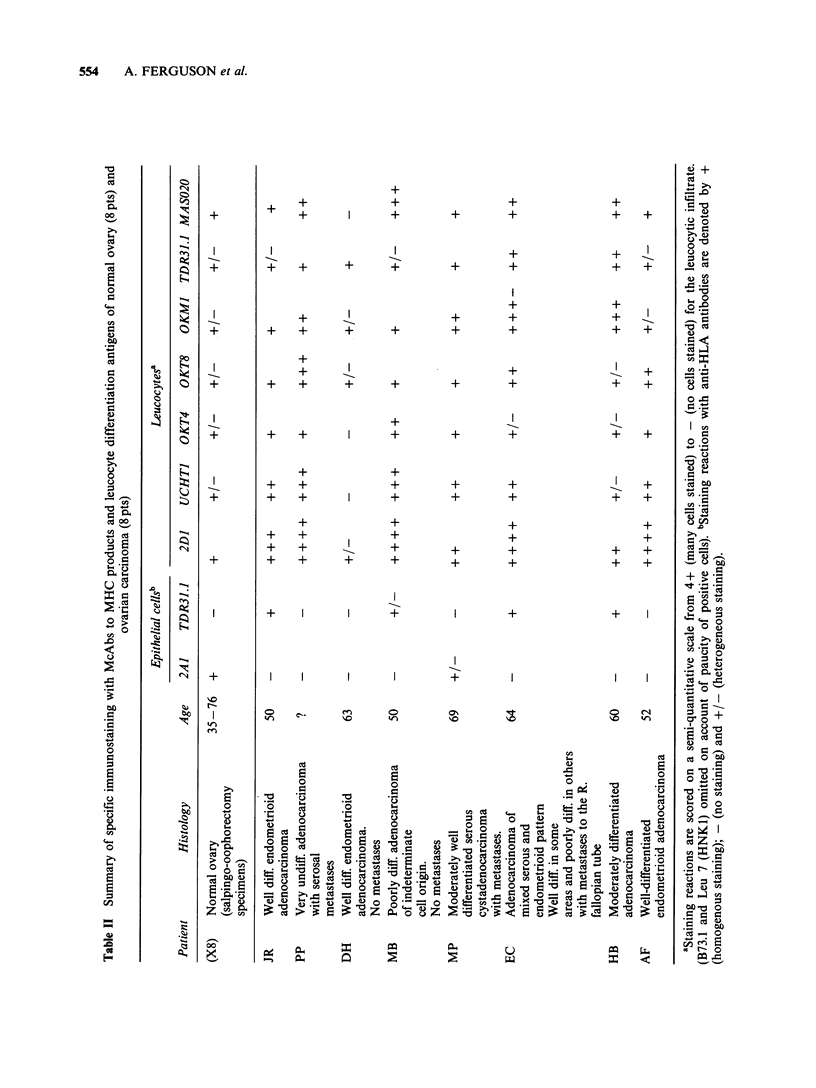

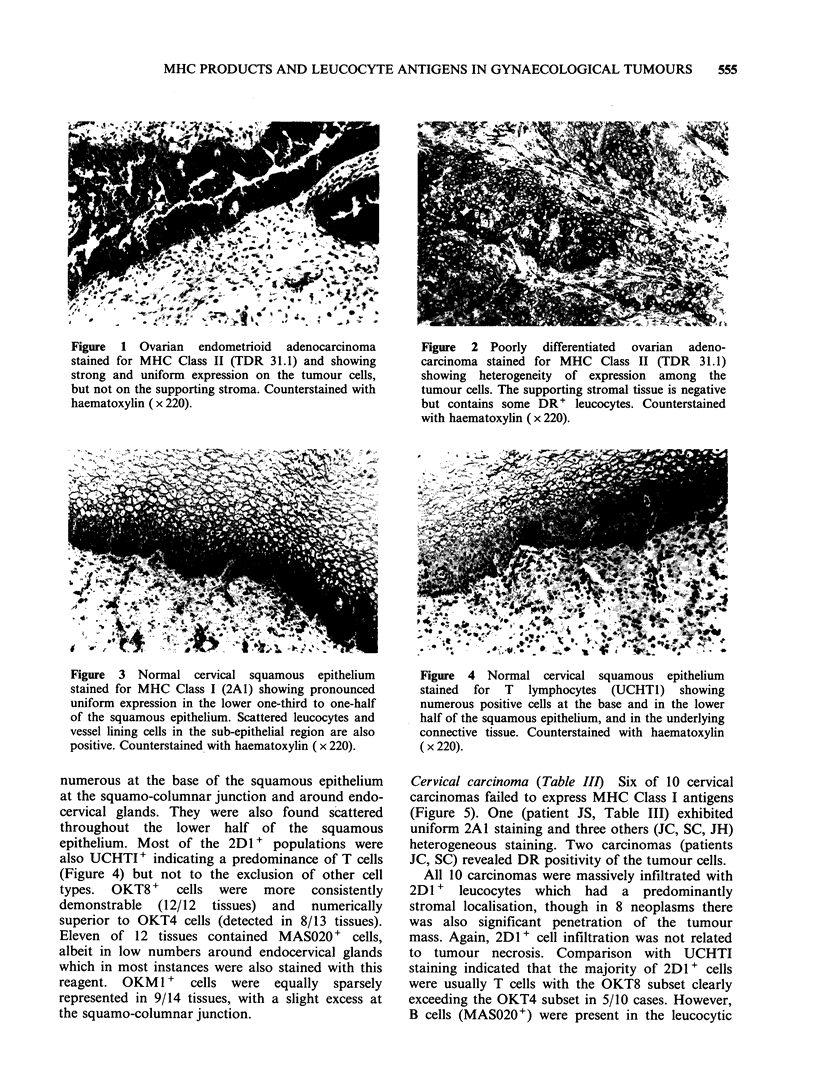

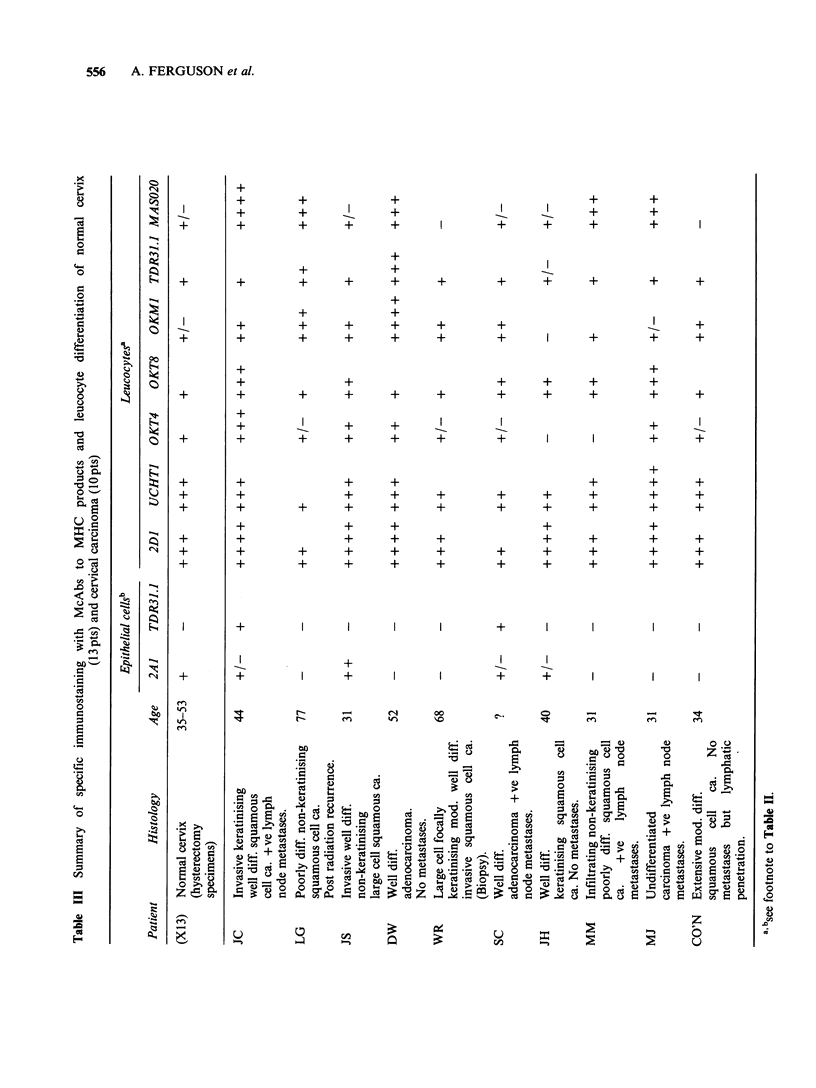

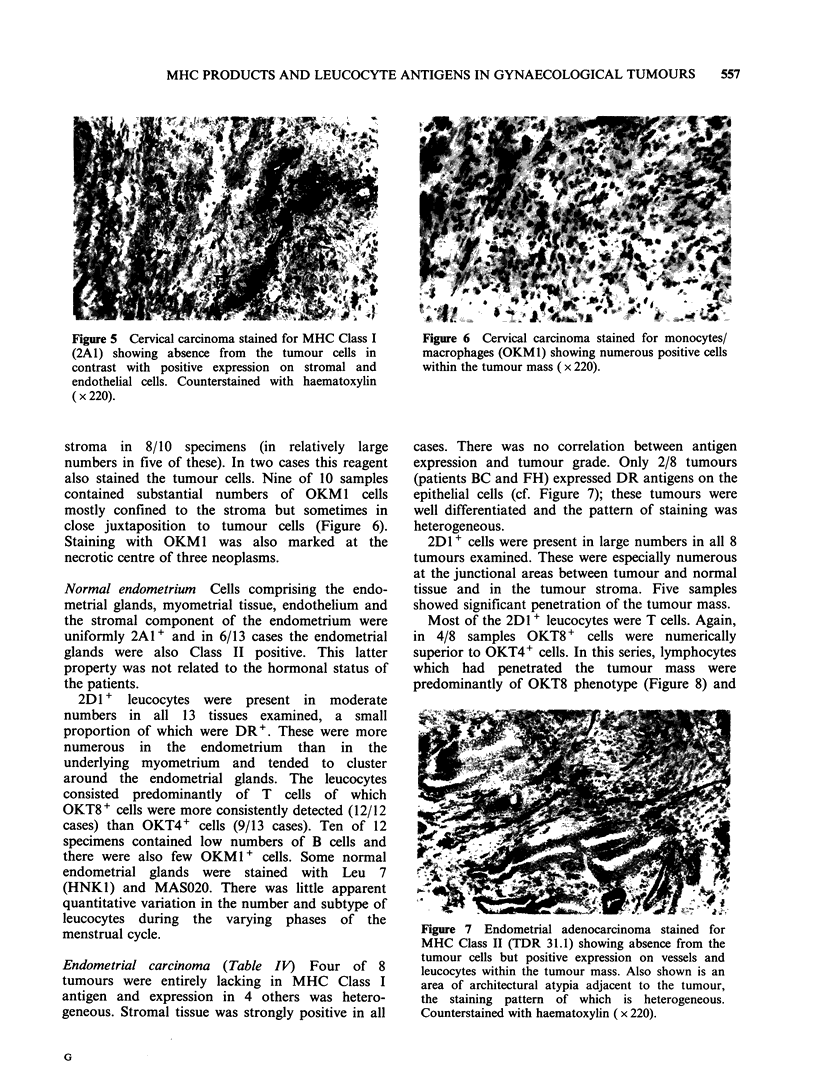

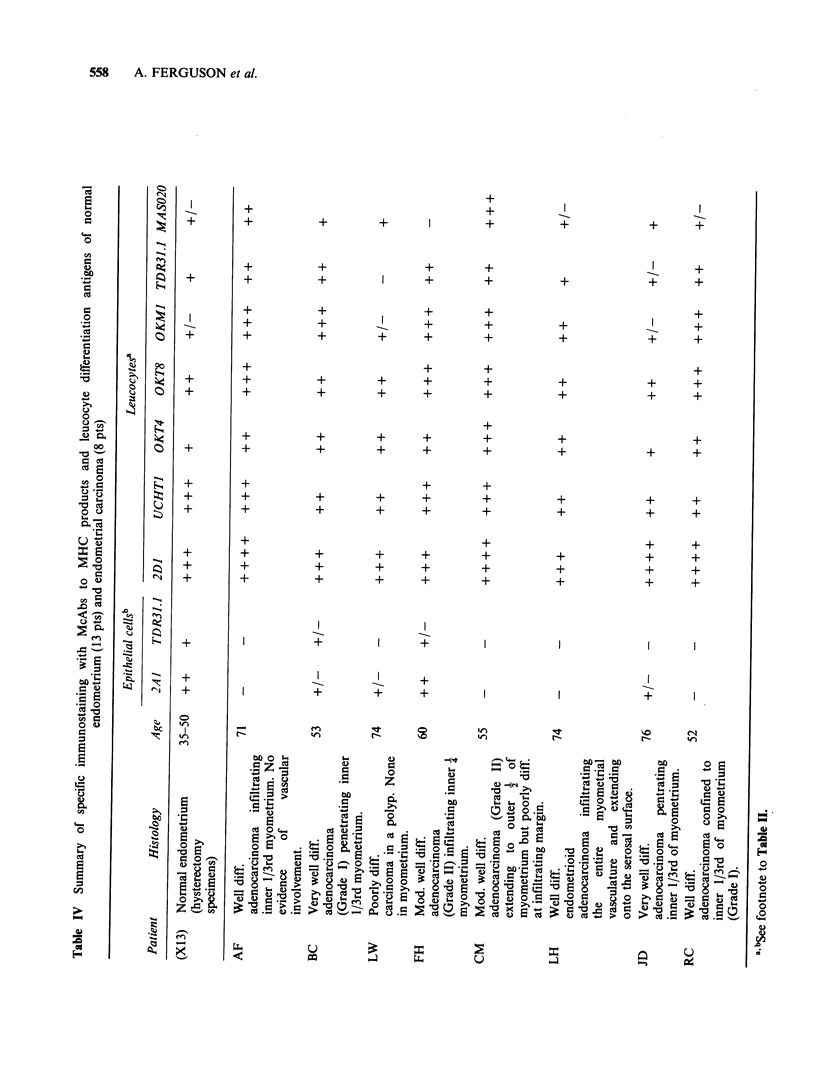

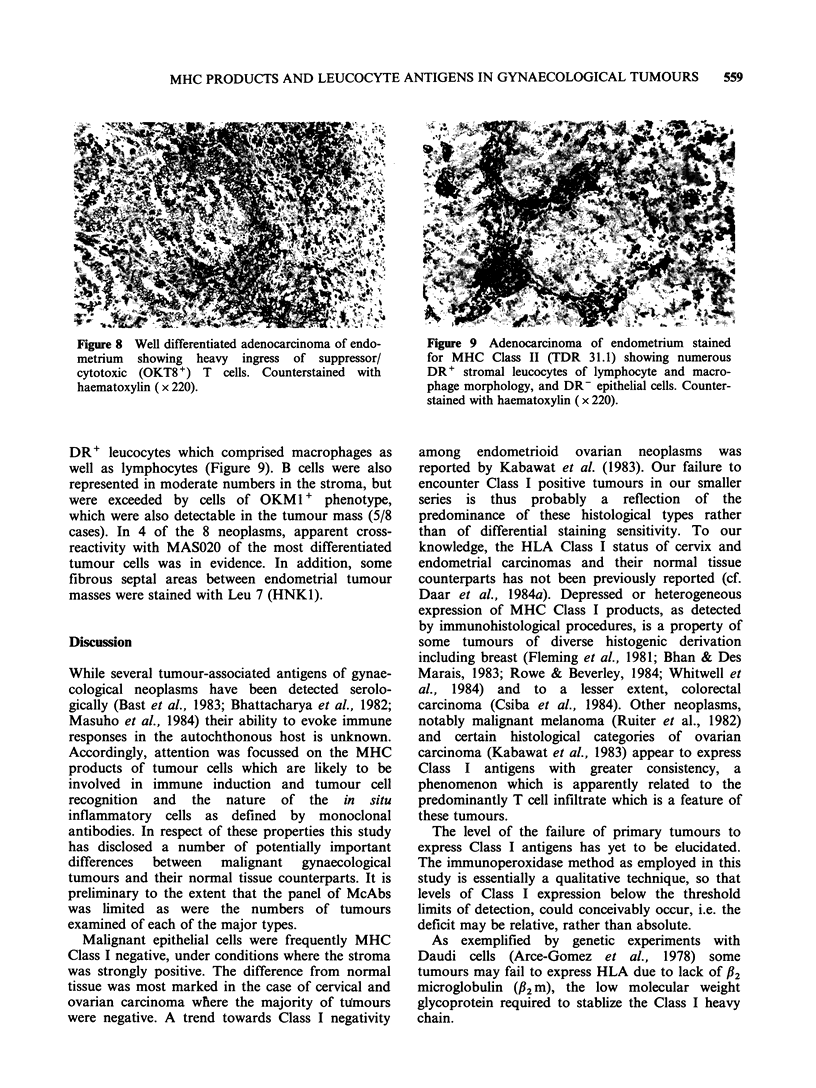

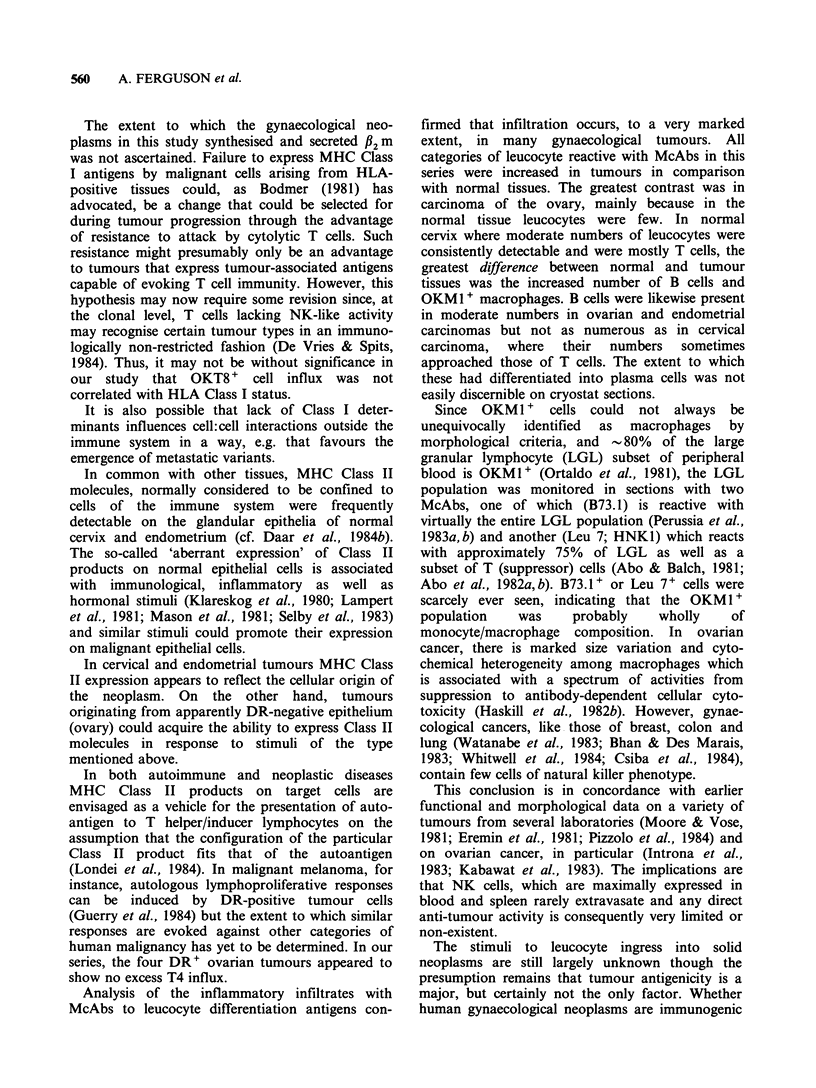

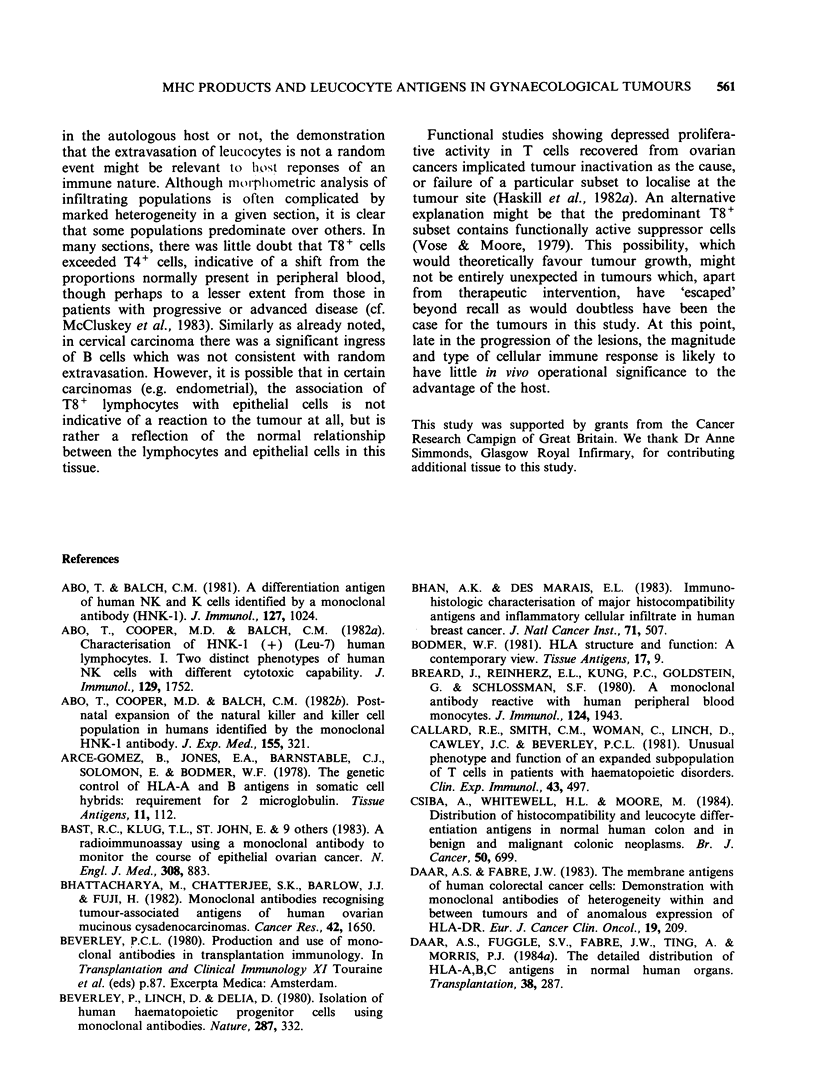

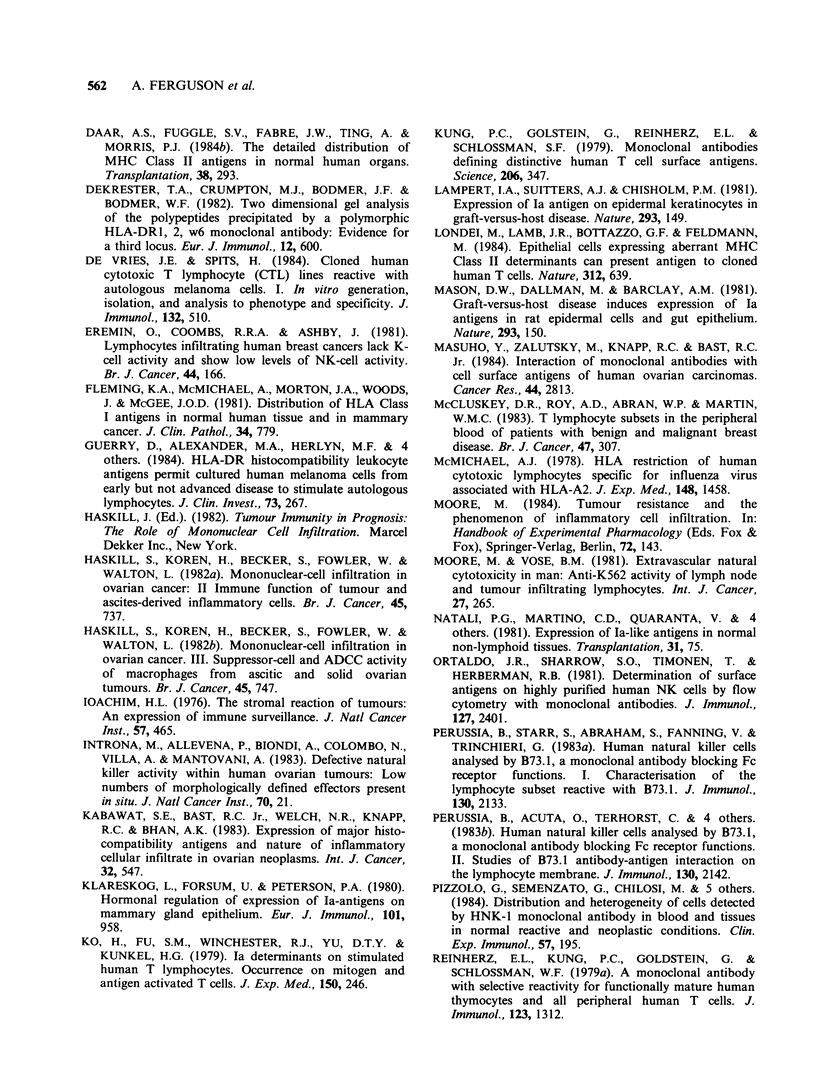

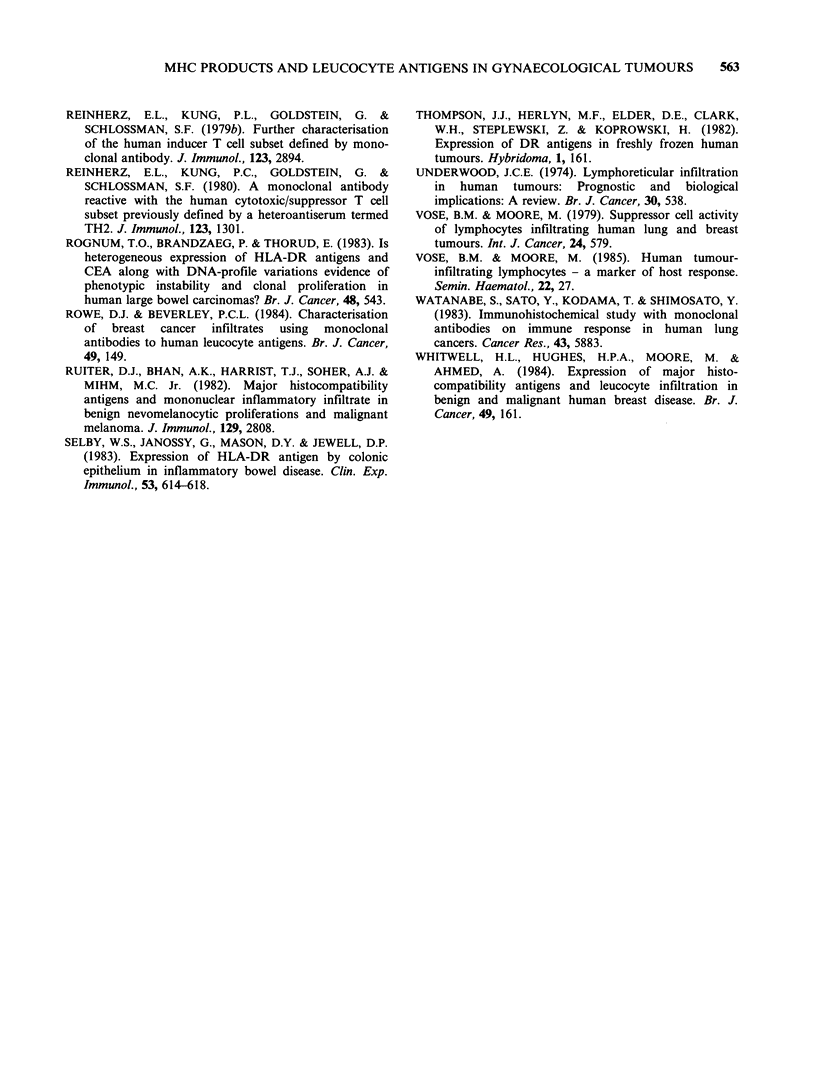


## References

[OCR_01397] Abo T., Balch C. M. (1981). A differentiation antigen of human NK and K cells identified by a monoclonal antibody (HNK-1).. J Immunol.

[OCR_01402] Abo T., Cooper M. D., Balch C. M. (1982). Characterization of HNK-1+ (Leu-7) human lymphocytes. I. Two distinct phenotypes of human NK cells with different cytotoxic capability.. J Immunol.

[OCR_01409] Abo T., Cooper M. D., Balch C. M. (1982). Postnatal expansion of the natural killer and keller cell population in humans identified by the monoclonal HNK-1 antibody.. J Exp Med.

[OCR_01415] Arce-Gomez B., Jones E. A., Barnstable C. J., Solomon E., Bodmer W. F. (1978). The genetic control of HLA-A and B antigens in somatic cell hybrids: requirement for beta2 microglobulin.. Tissue Antigens.

[OCR_01422] Bast R. C., Klug T. L., St John E., Jenison E., Niloff J. M., Lazarus H., Berkowitz R. S., Leavitt T., Griffiths C. T., Parker L. (1983). A radioimmunoassay using a monoclonal antibody to monitor the course of epithelial ovarian cancer.. N Engl J Med.

[OCR_01440] Beverley P. C., Linch D., Delia D. (1980). Isolation of human haematopoietic progenitor cells using monoclonal antibodies.. Nature.

[OCR_01445] Bhan A. K., DesMarais C. L. (1983). Immunohistologic characterization of major histocompatibility antigens and inflammatory cellular infiltrate in human breast cancer.. J Natl Cancer Inst.

[OCR_01428] Bhattacharya M., Chatterjee S. K., Barlow J. J., Fuji H. (1982). Monoclonal antibodies recognizing tumor-associated antigen of human ovarian mucinous cystadenocarcinomas.. Cancer Res.

[OCR_01451] Bodmer W. F. (1981). HLA structure and function: a contemporary view.. Tissue Antigens.

[OCR_01455] Breard J., Reinherz E. L., Kung P. C., Goldstein G., Schlossman S. F. (1980). A monoclonal antibody reactive with human peripheral blood monocytes.. J Immunol.

[OCR_01461] Callard R. E., Smith C. M., Worman C., Linch D., Cawley J. C., Beverley P. C. (1981). Unusual phenotype and function of an expanded subpopulation of T cells in patients with haemopoietic disorders.. Clin Exp Immunol.

[OCR_01468] Csiba A., Whitwell H. L., Moore M. (1984). Distribution of histocompatibility and leucocyte differentiation antigens in normal human colon and in benign and malignant colonic neoplasms.. Br J Cancer.

[OCR_01475] Daar A. S., Fabre J. W. (1983). The membrane antigens of human colorectal cancer cells: demonstration with monoclonal antibodies of heterogeneity within and between tumours and of anomalous expression of HLA-DR.. Eur J Cancer Clin Oncol.

[OCR_01482] Daar A. S., Fuggle S. V., Fabre J. W., Ting A., Morris P. J. (1984). The detailed distribution of HLA-A, B, C antigens in normal human organs.. Transplantation.

[OCR_01490] Daar A. S., Fuggle S. V., Fabre J. W., Ting A., Morris P. J. (1984). The detailed distribution of MHC Class II antigens in normal human organs.. Transplantation.

[OCR_01496] De Kretser T. A., Crumpton M. J., Bodmer J. G., Bodmer W. F. (1982). Two-dimensional gel analysis of the polypeptides precipitated by a polymorphic HLA-DR1,2,w6 monoclonal antibody: evidence for a third locus.. Eur J Immunol.

[OCR_01510] Eremin O., Coombs R. R., Ashby J. (1981). Lymphocytes infiltrating human breast cancers lack K-cell activity and show low levels of NK-cell activity.. Br J Cancer.

[OCR_01516] Fleming K. A., McMichael A., Morton J. A., Woods J., McGee J. O. (1981). Distribution of HLA class 1 antigens in normal human tissue and in mammary cancer.. J Clin Pathol.

[OCR_01522] Guerry D., Alexander M. A., Herlyn M. F., Zehngebot L. M., Mitchell K. F., Zmijewski C. M., Lusk E. J. (1984). HLA-DR histocompatibility leukocyte antigens permit cultured human melanoma cells from early but not advanced disease to stimulate autologous lymphocytes.. J Clin Invest.

[OCR_01534] Haskill S., Koren H., Becker S., Fowler W., Walton L. (1982). Mononuclear-cell infiltration in ovarian cancer. II. Immune function of tumour and ascites-derived inflammatory cells.. Br J Cancer.

[OCR_01541] Haskill S., Koren H., Becker S., Fowler W., Walton L. (1982). Mononuclear-cell infiltration in ovarian cancer. III. Suppressor-cell and ADCC activity of macrophages from ascitic and solid ovarian tumours.. Br J Cancer.

[OCR_01553] Introna M., Allavena P., Biondi A., Colombo N., Villa A., Mantovani A. (1983). Defective natural killer activity within human ovarian tumors: low numbers of morphologically defined effectors present in situ.. J Natl Cancer Inst.

[OCR_01548] Ioachim H. L. (1976). The stromal reaction of tumors: an expression of immune surveillance.. J Natl Cancer Inst.

[OCR_01560] Kabawat S. E., Bast R. C., Welch W. R., Knapp R. C., Bhan A. K. (1983). Expression of major histocompatibility antigens and nature of inflammatory cellular infiltrate in ovarian neoplasms.. Int J Cancer.

[OCR_01567] Klareskog L., Forsum U., Peterson P. A. (1980). Hormonal regulation of the expression of Ia antigens on mammary gland epithelium.. Eur J Immunol.

[OCR_01573] Ko H. S., Fu S. M., Winchester R. J., Yu D. T., Kunkel H. G. (1979). Ia determinants on stimulated human T lymphocytes. Occurrence on mitogen- and antigen-activated T cells.. J Exp Med.

[OCR_01579] Kung P., Goldstein G., Reinherz E. L., Schlossman S. F. (1979). Monoclonal antibodies defining distinctive human T cell surface antigens.. Science.

[OCR_01585] Lampert I. A., Suitters A. J., Chisholm P. M. (1981). Expression of Ia antigen on epidermal keratinocytes in graft-versus-host disease.. Nature.

[OCR_01590] Londei M., Lamb J. R., Bottazzo G. F., Feldmann M. (1984). Epithelial cells expressing aberrant MHC class II determinants can present antigen to cloned human T cells.. Nature.

[OCR_01596] Mason D. W., Dallman M., Barclay A. N. (1981). Graft-versus-host disease induces expression of Ia antigen in rat epidermal cells and gut epithelium.. Nature.

[OCR_01602] Masuho Y., Zalutsky M., Knapp R. C., Bast R. C. (1984). Interaction of monoclonal antibodies with cell surface antigens of human ovarian carcinomas.. Cancer Res.

[OCR_01608] McCluskey D. R., Roy A. D., Abram W. P., Martin W. M. (1983). T lymphocyte subsets in the peripheral blood of patients with benign and malignant breast disease.. Br J Cancer.

[OCR_01614] McMichael A. (1978). HLA restriction of human cytotoxic T lymphocytes specific for influenza virus. Poor recognition of virus associated with HLA A2.. J Exp Med.

[OCR_01625] Moore M., Vose B. M. (1981). Extravascular natural cytotoxicity in man: Anti-K562 activity of lymph-node and tumour-infiltrating lymphocytes.. Int J Cancer.

[OCR_01636] Ortaldo J. R., Sharrow S. O., Timonen T., Herberman R. B. (1981). Determination of surface antigens on highly purified human NK cells by flow cytometry with monoclonal antibodies.. J Immunol.

[OCR_01651] Perussia B., Acuto O., Terhorst C., Faust J., Lazarus R., Fanning V., Trinchieri G. (1983). Human natural killer cells analyzed by B73.1, a monoclonal antibody blocking Fc receptor functions. II. Studies of B73.1 antibody-antigen interaction on the lymphocyte membrane.. J Immunol.

[OCR_01643] Perussia B., Starr S., Abraham S., Fanning V., Trinchieri G. (1983). Human natural killer cells analyzed by B73.1, a monoclonal antibody blocking Fc receptor functions. I. Characterization of the lymphocyte subset reactive with B73.1.. J Immunol.

[OCR_01658] Pizzolo G., Semenzato G., Chilosi M., Morittu L., Ambrosetti A., Warner N., Bofill M., Janossy G. (1984). Distribution and heterogeneity of cells detected by HNK-1 monoclonal antibody in blood and tissues in normal, reactive and neoplastic conditions.. Clin Exp Immunol.

[OCR_01680] Reinherz E. L., Kung P. C., Goldstein G., Schlossman S. F. (1980). A monoclonal antibody reactive with the human cytotoxic/suppressor T cell subset previously defined by a heteroantiserum termed TH2.. J Immunol.

[OCR_01665] Reinherz E. L., Kung P. C., Goldstein G., Schlossman S. F. (1979). A monoclonal antibody with selective reactivity with functionally mature human thymocytes and all peripheral human T cells.. J Immunol.

[OCR_01674] Reinherz E. L., Kung P. C., Goldstein G., Schlossman S. F. (1979). Further characterization of the human inducer T cell subset defined by monoclonal antibody.. J Immunol.

[OCR_01687] Rognum T. O., Brandtzaeg P., Thorud E. (1983). Is heterogeneous expression of HLA-dr antigens and CEA along with DNA-profile variations evidence of phenotypic instability and clonal proliferation in human large bowel carcinomas?. Br J Cancer.

[OCR_01693] Rowe D. J., Beverley P. C. (1984). Characterisation of breast cancer infiltrates using monoclonal antibodies to human leucocyte antigens.. Br J Cancer.

[OCR_01699] Ruiter D. J., Bhan A. K., Harrist T. J., Sober A. J., Mihm M. C. (1982). Major histocompatibility antigens and mononuclear inflammatory infiltrate in benign nevomelanocytic proliferations and malignant melanoma.. J Immunol.

[OCR_01706] Selby W. S., Janossy G., Mason D. Y., Jewell D. P. (1983). Expression of HLA-DR antigens by colonic epithelium in inflammatory bowel disease.. Clin Exp Immunol.

[OCR_01712] Thompson J. J., Herlyn M. F., Elder D. E., Clark W. H., Steplewski Z., Koprowski H. (1982). Expression of DR antigens in freshly frozen human tumors.. Hybridoma.

[OCR_01718] Underwood J. C. (1974). Lymphoreticular infiltration in human tumours: prognostic and biological implications: a review.. Br J Cancer.

[OCR_01728] Vose B. M., Moore M. (1985). Human tumor-infiltrating lymphocytes: a marker of host response.. Semin Hematol.

[OCR_01723] Vose B. M., Moore M. (1979). Suppressor cell activity of lymphocytes infiltrating human lung and breast tumours.. Int J Cancer.

[OCR_01733] Watanabe S., Sato Y., Kodama T., Shimosato Y. (1983). Immunohistochemical study with monoclonal antibodies on immune response in human lung cancers.. Cancer Res.

[OCR_01739] Whitwell H. L., Hughes H. P., Moore M., Ahmed A. (1984). Expression of major histocompatibility antigens and leucocyte infiltration in benign and malignant human breast disease.. Br J Cancer.

[OCR_01503] de Vries J. E., Spits H. (1984). Cloned human cytotoxic T lymphocyte (CTL) lines reactive with autologous melanoma cells. I. In vitro generation, isolation, and analysis to phenotype and specificity.. J Immunol.

